# A clinical approach to the investigation and management of long COVID associated neuropathic pain

**DOI:** 10.1007/s00406-023-01721-8

**Published:** 2023-12-08

**Authors:** Rajish Sanjit Kumar Shil, Thomas William Hughes, Brendan Francis Sargent, Yun Huang, Arina Anna Tamborska, Bernhard Frank, Mark Alexander Ellul, Benedict Daniel Michael

**Affiliations:** 1 Clinical Infection, Microbiology and Immunology, Institute of Infection, Veterinary and Ecological Sciences, University of Liverpool, Liverpool, UK; 2 Department of Neurology, Walton Centre of Neurosurgery and Neurology, Liverpool, UK; 3 Liverpool University Hospitals NHS Foundation Trust, Liverpool, UK; 4 Nuffield Department of Clinical Neurosciences, University of Oxford, John Radcliffe Hospital, Oxford, UK; 5 Department of Psychiatry, University of Oxford, Oxford, UK; 6 National Institute for Health Research Health Protection Research Unit in Emerging and Zoonotic Infections, Institute of Infection, Veterinary and Ecological Sciences, University of Liverpool, Liverpool, UK; 7 Department of Pain Medicine, Walton Centre of Neurosurgery and Neurology, Liverpool, UK; 8 Faculty of Health and Life Sciences, Pain Research Institute, University of Liverpool, Liverpool, UK

**Keywords:** Long Covid, Neuropathy, Pain, Paraesthesia, COVID-19

## Abstract

COVID–19 has been associated with a wide range of ongoing symptoms following recovery from the acute SARS-CoV-2 infection. Around one in three people with COVID-19 develop neurological symptoms with many reporting neuropathic pain and associated symptoms, including paraesthesia, numbness, and dysesthesia. Whilst the pathophysiology of long COVID-19-associated neuropathic pain remains unclear, it is likely to be multifactorial. Early identification, exclusion of common alternative causes, and a biopsychosocial approach to the management of the symptoms can help in relieving the burden of disease and improving the quality of life for patients.

## Introduction

### Neurological manifestations of long COVID

COVID-19 is associated with a wide range of persisting symptoms following resolution of the acute infection with SARS-CoV-2, which are collectively termed as ‘post-acute sequelae of SARS-CoV-2 infection’ (PASC) or more colloquially as ‘long COVID’ [1–5]. Long COVID presents with clusters of symptoms, which are overlapping and multisystemic, are not explained by an alternative diagnosis, and can last from between 4 and 12 weeks (classified as ongoing symptomatic COVID-19) to up to more than 12 weeks (classified as post-COVID-19 syndrome) [6]. This is distinct from patients who have suffered recognised neurological diagnoses during acute SARS-CoV-2 infection such as cerebrovascular events, demyelination, and encephalitis [7–9]. Symptoms of long COVID involve multiple organ systems, including respiratory (cough and dyspnoea), psychiatric (anxiety, depression, and irritability), cardiac (e.g., palpitations), and gastrointestinal (e.g. nausea). Associated neurological symptoms include myalgia, fatiguability, headache, anosmia, dizziness, cognitive disorder, sleep disorder, and neuropathic symptoms such as paraesthesia and pain [10].

### Neuropathic pain post-COVID

Neuropathic pain following COVID-19 infection has been reported as burning pain, painful cold sensations, pins and needles sensations, itchiness, electric shock-like sensations, hypoesthesia, and dysesthesia. These symptoms can be distributed across various parts of the body, including limbs, trunk, neck, and less commonly reported as ‘burning eyes or mouth syndrome’, which might not present to neurologists [11]. These symptoms can be seen in acute COVID-19 but are reported more commonly in the context of long COVID, particularly within the first 6 months after the onset of acute COVID illness [12]. Up to 45% of the patients with COVID-19, regardless of hospitalisation status, develop long COVID symptoms [13]. Neuropathic pain has been reported to affect approximately one in nine patients with long COVID, consequently, this may be suffered by a large number of people given the millions of patients affected by COVID-19 during the pandemic [14].

## Pathophysiology

The pathophysiology of neuropathic symptoms related to COVID-19 is unclear but may reflect infectious and para-infectious processes. Neuropathic pain in PASC has been hypothesized to be due to systemic inflammation from immune dysregulation, neuroinflammation, neuronal injury leading to demyelination, or stroke due to vascular dysfunction leading to occlusion or cerebral microbleeds [15–17]. Small-fibre neuropathy has been reported as the most common in patients with long COVID without any alternative aetiology identified [12]. In common with chronic fatigue syndrome or myalgic encephalomyelitis, another multisystem neuroimmunology disorder often preceded by infection, patients with long COVID are often erroneously diagnosed with psychiatric disorders which can delay access to effective treatment. Mental health disorders may co-exist as either a risk factor or consequence of long COVID, but the emerging evidence supports an organic, post-viral component to the condition [15]. Neuropathic pain may occur indirectly as a result of prolonged hospital admission, particularly if care in the intensive treatment unit (ITU) is required, where patients are at risk of developing critical illness neuropathy [18].

## Symptomatology

### Neuropathic pain symptoms associated with long COVID

Neuropathic pain is pain caused by a lesion or disease of the somatosensory nervous system as defined by the International Association for the Study of Pain [19, 20]. Neuropathic pain is described as spontaneous burning, electrical, or shooting pain by patients who may also report a squeezing, cold, or itchy sensation. The clinical examination may show reduced pin prick or thermal sensation, allodynia, (pain caused by a painless stimulus), and hyperalgesia (exaggerated painful sensation with a normal pain stimulus).

A review by Joshi et al. reported patients presenting with neuropathic pain post COVID in multiple locations, ranging from generalized burning sensation throughout the body, to scorching pain in the extremities, isolated upper limb symptoms, neck and back pain ranging from C1 to L5, cranial nerve involvement as trigeminal neuralgia, and few cases of herpetic neuralgiform pain occurring in the context of SARS-CoV-2 infection [21]. There are also a few cases reported of complex regional pain syndrome following acute COVID-19 illness, presenting as asymmetrical limb weakness associated with pain, hyperalgesia, allodynia, swelling, and deformities, requiring a multi-disciplinary treatment approach [22, 23].

### Clinical Classification of neuropathic pain following acute COVID-19

Clinical classification of COVID-19-associated neuropathic pain has been proposed including five different categories: pain following ITU admission due to COVID-19, pain following a COVID-19-associated stroke, pain attributable to COVID-19-associated transverse myelitis and Guillain-Barré syndrome (GBS), and chronic neuropathic pain due to infection with SARS-CoV-2 without another clinical diagnosis [24].

Following ITU admission with COVID-19, persistent pain can be associated with joint contractures or muscle atrophy, critical illness myopathy, or polyneuropathy. This can be attributed to pressure on specific nerves during prone positioning for the management of acute respiratory distress syndrome (ARDS) or prolonged lying in the supine position while receiving neuromuscular blocking agents [25].

In patients with neuropathic symptoms following COVID-19 who did not require hospitalisation or ITU admission, this may be due to direct [26] or indirect nerve injury, to host inflammatory processes, or unmasking of pre-existing neuropathy (e.g. diabetic, alcohol, iatrogenic), or central sensitisation driven by secondary hyperalgesia to mechanical stimuli [27]. In those patients who suffered a cerebrovascular event in the context of acute SARS-CoV-2 infection, well-established mechanisms, such as thalamic infarction, can result in central pain syndromes, which can be particularly difficult to treat [28].

Neuropathic pain can also develop due to transverse myelitis associated with COVID-19 with symptoms at or below the level of the lesion [29–33]. GBS has been reported in patients who have recently had COVID-19, including in older persons, which may or may not be proportionate to the severity of the respiratory illness [34]. GBS associated with neuropathic pain occurs, mainly via impairment of small nociceptive fibres [35] and there have been several reports of chronic neuropathic pain and myalgia in severe cases of GBS associated with COVID-19 [34, 36].

In patients where an alternate diagnosis is not present for neuropathic pain, the diagnosis of small-fibre neuropathy was suggested as a cause of the pain, and this could be potentially challenging to treat as reported in a few cases [21, 37, 38].

### Neuropathic pain developing in the convalescent period

The time course of the development of pain is variable following COVID-19 infection. In one study, the pain symptoms increased in time up to one year post infection [39]. There has been no evidence that patients with pre-existing neuropathy routinely developed worsening of their symptoms following COVID-19. However, the pandemic might have had an indirect impact, for example, due to missed or delayed follow-up, patients running out of their usual pain medications, and ongoing psychological distress [40].

## Diagnostic evaluation

Diagnostic evaluation for long COVID-related neuropathic pain needs to be tailored to the clinical presentation. If patients have pre-existing neuropathy-related pain or chronic pain syndromes, this too needs to be evaluated. Whilst neuropathic symptoms have been reported following COVID-19, clinicians need to be mindful to exclude other common, reversible, or serious aetiologies in patients presenting following COVID-19 ([Table 1). To support this, grading systems to both identify neuropathic pain in clinical and research settings and to aid in appropriate diagnostic evaluation and prompt treatment consideration can often be useful [41]. There is a need for uniform definitions for neurological syndromes associated with COVID-19, which can also aid in diagnostic categorisation [42].

**Table 1:** Causes of neuropathic pain following COVID-19 in comparison to other causes

Causes of neuropathy	Examples
*COVID-19-related Neuropathic Pain*	
Compression Neuropathy	History of proning in COVID-19 patients for treatment of ARDS in ITU, symptom distribution to specific nerves e.g., ulnar, radial or peroneal neuropathy
Critical illness neuropathy	Severe COVID-19 requiring hospitalization ± intensive care; axonal sensorimotor peripheral neuropathy on nerve conduction studies
Drug related	Neurotoxic drugs used for acute COVID-19 illness (daptomycin, linezolid, lopinavir, ritonavir, hydroxychloroquine, cisatracurium, clindamycin, glucocorticoids)
Stroke/Inflammatory	Thalamic pain secondary to stroke, Pain secondary to inflammation e.g., transverse myelitis and Gullian Barre Syndrome
*Other causes to be considered/pre-existing*	
Metabolic/Nutritional	Diabetes Mellitus, Hypothyroidism, Uraemia, B12/B1 deficiency
Malignancy	Paraneoplastic syndromes, paraprotein associated (POEMS syndrome, myeloma, secondary amyloidosis)
Infectious	Hepatitis, HIV, Syphilis, Leprosy, Lyme disease (where clinically indicated)
Inflammatory/Autoimmune	Chronic inflammatory demyelinating polyneuropathy, Vasculitis like granulomatosis with polyangiitis, mononeuritis multiplex, Sarcoidosis, SLE, Sjogren’s syndrome, Rheumatoid arthritis
Radiculopathy	Degenerative disc disease, trauma
Drugs/Toxins	Antiretroviral treatment, chemotherapeutic agents, amiodarone, lithium, alcohol excess, heavy metals e.g., lead, and nitrous oxide poisoning
Hereditary	Acute intermittent porphyria, Hereditary sensory-motor neuropathy, Fabry’s disease

Abbreviations: *ARDS* Acute respiratory distress syndrome, *ITU* intensive treatment unit, *POEMS* Polyneuropathy, organomegaly, endocrinopathy, monoclonal gammopathy, skin abnormalities, *HIV* Human immunodeficiency virus, *SLE* systemic lupus erythematosus

**Fig. 1:**
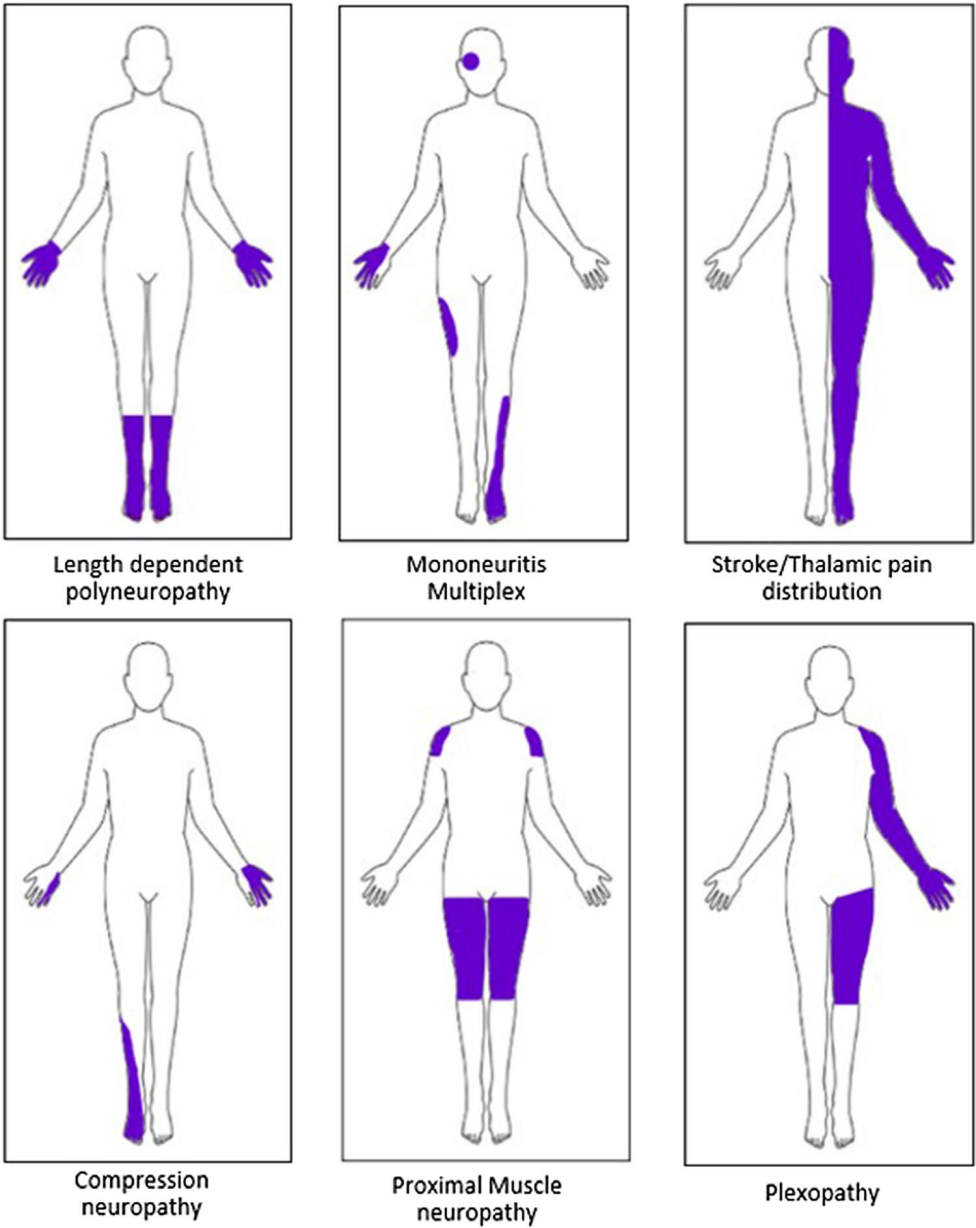
Accompaniment of Table 2. No specific pattern of pain distribution in long COVID associated neuropathic pain, as can be a combination of the above patterns

Within the clinical history, it is important to assess for neoplastic red flag symptoms, including weight loss, lymphadenopathy, and symptoms of anaemia, alongside organ-specific symptoms (such as haemoptysis and melaena, and associated risk factors (e.g. smoking). A detailed social history should include alcohol and recreational drug use (e.g. nitrous oxide inhalation). The physical examination of the peripheral nerves should include an assessment by inspection for skin changes and ulcers, pes cavus and claw toes, wasting of the distal muscles, tremors, and pseudo athetosis, and assessment for sensory loss distribution (including light touch, pinprick, proprioception, and vibration sense, at the fingertips). Assess the pattern of muscle weakness to determine whether length-dependent neuropathy is distal and symmetrical, or polyradiculoneuropathy which is non-length-dependent [43]. The pattern of neuropathic symptoms associated with COVID-19 is a comparative mixture of symptoms related to other specific causes of neuropathy which may or may not be present with more typical neuropathic signs (Table 2, Fig. 1).

**Table 2:** Pattern of symptoms and signs of neuropathy along with potential investigations

Neuropathy	Symptoms/signs	Potential investigations
Long COVID-19 associated neuropathy	Numbness, paraesthesia, burning pain, tingling	COVID-19 PCR positive (recent)
Length-dependent small fibre neuropathy	Mostly length dependent as stocking or glove-stocking pattern, burning pain, numbness, electric shock-like pain, worse at night. ± autonomic dysfunction	NCS: Often normal Serum ± urine investigations for secondary causes
Large fibre neuropathy	Sensory ataxia, numbness, muscle weakness, loss of vibration and proprioception, reduced reflexes	NCS: Abnormal Investigate for secondary causes
Neuronopathies	Early onset ataxia and asymmetric non-length-dependent or generalized sensory deficits	NCS: Abnormal Vitamin B6 level (Toxicity) Vasculitis: ANA, Anti SSA, AntiDS-DNA Paraneoplastic: antiHu, antiCV2, CRMP5, CT chest abdomen pelvis, PET-CT, Biopsy
Neuromyopathy	A critical illness-related, frequent complication of ITU, both motor and sensory symptoms	NCS/EMG: Axonal sensorimotor peripheral neuropathy
Mononeuritis multiplex	Numbness, weakness, pain, presence of risk factors	NCS/EMG, HbA1c, vasculitis workup, viral workup, ACE, paraneoplastic screen
AIDP/CIDP	AIDP: Ascending paralysis, areflexia, motor, and sensory involvement CIDP: Relapsing course, proximal and distal weakness, sensory-motor involvement	NCS: Demyelinating pattern LP: Albumino-cytologic ratio Rule out concomitant diseases e.g., Diabetes, MGUS, HIV, Hepatitis, SLE, Sarcoidosis, Thyroid diseases, Lyme disease (where clinically indicated)

Abbreviations: *PCR* Polymerase chain reaction, *NCS* Nerve conduction studies, *LP* Lumbar Puncture, *ANA* Antinuclear antibodies, *Anti-SSA* Anti Sjogren’s syndrome-related antigen A, *Anti-DS-DNA* anti-double-stranded DNA, *CRMP5* Collapsin receptor mediator 5, *CT* Computed tomography, *PET-CT* Positron emission tomography – computed tomography, *EMG* Electromyography, *AIDP* Acute inflammatory demyelinating polyneuropathy, *CIDP* Chronic inflammatory demyelinating polyneuropathy, *MGUS* Monoclonal gammopathy of undetermined significance, *HIV* Human immunodeficiency virus, *SLE* Systemic lupus erythematosus

Consider a referral to a neurologist for assessment and further specialised investigations, if there are features of complex neurological disease in the form of central nervous system involvement, ataxia, progressive symptoms, weakness, wasting, fasciculations, or marked loss of proprioception, and/or vibration, or asymmetry.

## Investigations

Laboratory Investigations should include blood tests for common, reversible, or sinister aetiologies: Full blood count, renal function, liver function, erythrocyte sedimentation rate, C-reactive protein, vitamin B12, folate, blood glucose, HbA1c, creatine kinase, thyroid function, and serum protein electrophoresis [Table 2]. These tests would essentially rule out haematological, metabolic, autoimmune, nutritional, and endocrine causes of neuropathy, like diabetes and hypothyroidism. In cases with a large fibre-predominant pattern (disproportionate loss of proprioception and vibration) or those with gastrointestinal or dietary risk factors consider testing methylmalonic acid and homocysteine to evaluate for cellular B12 deficiency even in the presence of a normal or near normal B12 level. In those with disproportionate sensory ataxia and/or early upper limb involvement consider evaluation for a neuronopathy. In those with painful, and/or asymmetric, and or progressive features, also evaluate for vasculitis and paraneoplastic pathology, to rule out potentially life-threatening conditions [43]. Based on the social history of the patients, it is worth to consider investigation for any toxins or heavy metals ingestion from occupational exposure, nitrous oxide from recreational drug use, and for any malabsorption syndromes based on nutritional history, all of which can lead to neuropathy related symptoms.

Specialised investigations, including electromyography and nerve conduction studies, are reported to be normal in most cases of long COVID-19-associated neuropathy, though several reports have identified axonal, demyelinating, and/or mixed patterns of conduction abnormalities [44]. A skin or nerve biopsy to assess small nerve fibre may be helpful if an alternative aetiology (especially vasculitic or infiltrative) is suspected and has also been reported as abnormal in some cases associated with COVID-19 with reduced nerve fibre density, along with vascular and perineural changes consistent with nerve-ending hypertrophy [45, 46]. However, this is not widely available and any role in evaluating neuropathic symptoms which are suspected to be due to COVID-19 has not been established. A lumbar puncture is indicated when an inflammatory aetiology is suspected, such as GBS, to evaluate the cerebrospinal fluid for protein, white cell counts along with oligoclonal bands, and cytology when demyelination or malignancy are suspected respectively. Magnetic resonance imaging of the brain and/or spine are indicated when symptoms and signs suggest a central cause, such as hemisensory patterns seen in COVID-associated stroke or parasensory/motor symptoms and signs of COVID-19-associated myelitis.

In addition to the above extensive investigations, it is also essential to consider the psychological aspects of the symptoms, including evaluating any history of psychiatric comorbidities, which were shown to be an independent predictor of long-COVID symptoms [47].

## Treatment recommendations

Treatment strategy for neuropathic pain associated with Long COVID-19 follows the same approaches as used for managing neuropathic pain from other causes. Early detection of the causes of neuropathic pain due to COVID-19 and initiation of appropriate evidence-based treatment may also further reduce other symptoms of long COVID such as fatigue and cognitive symptoms [16]. Multidisciplinary team (MDT) input is the most effective approach to the management of neuropathic pain and is recommended in multiple guidelines [48, 49]. The MDT approach also involves working closely with patients to improve their quality of life, in addition to physiotherapy, occupational therapy, and psychological therapy, to address issues such as pain, depression, anxiety, and sleep disturbances, in addition to alternative therapies, such as massage and acupuncture in some.

These nonpharmacological therapies are recommended to be tried for a duration of six to eight weeks if used alone. The next approach is to initiate the first line of pharmacological treatments if adequate pain relief is not obtained with conservative methods. However, the nonpharmacological measures are recommended to be continued while a patient is started on pharmacotherapy [46]. The analgesic treatment pathway for neuropathic pain differs from original world health organisation (WHO) analgesic ladder as adjuvant treatment like tricyclic antidepressants (TCA), gabapentinoids, and serotonin-norepinephrine reuptake inhibitors (SNRIs) are considered to be first-line treatments for an average of four to six weeks [50]. These medications can be started at a low dose and titrated based on pain-relieving effects and tolerability. Most of the treatment can be initiated in primary care with onward referral to a specialist pain clinic considered for those in whom these approaches have been unsuccessful. Chronic pain services often offer advanced treatment options, which may include off-label drug use, hospital-only treatments like botox and capsaicin patches, and neuromodulation in very selected patients, alongside the group and individualised psychological and physical rehabilitation.

Second-line pharmacological therapies include topical lidocaine plasters and topical high-concentration capsaicin for patients with focal neuropathic pain not exceeding the area which can be covered, especially in those who do not tolerate oral medications or wish to avoid them for other reasons such as co-morbidities or polypharmacy [49]. Third-line treatment includes opioids such as tramadol (for up to two weeks) and tapentadol. However, these are often considered first-line treatments for acute neuropathic pain, cancer-related pain, and breakthrough neuropathic pain. Tapentadol can be titrated, under specialist supervision, with the effectiveness measured as the degree of improved function and quality of life. Low-dose opioid treatment can also be considered under specialist supervision, however, given the lack of long-term efficacy data, significant side effect profile in multiple studies and risks of opioid dependency, it is recommended to try at a low dose and for a limited duration, with careful monitoring of the pain reduction and improvement in function as well as side effects like respiratory depression and constipation [51]. Alternatively, a combination of two first-line drugs can be trialled for patients not responding to a single agent [48].

When a specific neurological disease entity is diagnosed in a patient in the context of COVID-19, it is reasonable, to begin with the standard management approach as one would for non-COVID-19 patients. For example, in a series of 42 patients with a mixture of polyneuropathies in association with COVID-19 consistent with AIDP and CIDP who were treated with standard recommended treatment (intravenous immunoglobulins, plasma exchange, and methylprednisolone), this resulted in good clinical outcome [52].

Sometimes more exploratory approaches for pain relief have been described, such as epidural injections, pulse radiofrequency, adhesiolysis, sympathetic blocks, radiofrequency denervation, transcutaneous electrical nerve stimulation, and spinal cord stimulation; although the evidence base, particularly for COVID-19 is lacking and specialist input should be sought [53].

## Conclusion and key points

Neuropathic pain associated with COVID-19 has been widely reported in the context of well-established neurological diagnoses, such as stroke and demyelination, and also often in patients with long COVID.

- The first step in evaluation is to refine the nature and distribution of the sensory symptoms and signs alongside evaluation for any associated motor or autonomic features.
- Following this, the investigation should be directed towards identifying well-established neurological diagnoses which have been identified in patients at or around the time of SARS-CoV-2 infection, including cerebrovascular events, myelitis, and GBS.
- If these are not present, then a focused and sequential investigatory approach is important to exclude causes of neuropathic pain unrelated to COVID-19, such as diabetes, toxic/ metabolic/ iatrogenic/ hormonal pathologies, and progressive syndromes, including vasculitis, haematological disorders, and paraneoplastic diseases.
- If no specific neurological diagnosis or alternative cause is identified, then a patient-focused MDT approach with a tiered escalation in therapy which engages a biopsychosocial approach and early involvement of pain management specialists is appropriate.

Ultimately, further research is required to better refine the clinical phenotypes of neuropathic pain in long COVID and the associated underlying pathophysiology, which is likely to be heterogeneous, if we are to work towards ever-improved care for our patients.
